# Serum microRNA let-7a-1/let-7d/let-7f and *miRNA 143/145* Gene Expression Profiles as Potential Biomarkers in HCV Induced Hepatocellular Carcinoma

**DOI:** 10.31557/APJCP.2020.21.2.555

**Published:** 2020

**Authors:** Doaa Mamdouh Aly, Nadida Abdul- Hameed Gohar, Afaf Ahmed Abd El-Hady, Marwa Khairy, Mona Mohsen Abdullatif

**Affiliations:** 1 *Clinical and Chemical Pathology Department, Theodor Bilharz Research Institute, *; 2 *Department of Clinical and Chemical Pathology, *; 3 *Endemic Medicine and Hepatology Unit, Faculty of Medicine, Cairo University, Egypt. *

**Keywords:** Hepatocellular carcinoma, hepatitis C virus, microRNA, PCR

## Abstract

**Background:**

Egypt has the highest prevalence of hepatitis C virus (HCV) worldwide. Which make liver cirrhosis and hepatocellular carcinoma (HCC) major health concerns in Egypt. Circulating microRNAs (miRNAs) have been investigated as biomarkers for malignancies. We investigated miRNA gene expression of *Lethal-7 (let-7)* cluster: *let7-a-1*, *let-7d-1*, *let-7f-1* and *miRNA (miR)143/145* cluster in sera of HCC patients and chronic HCV patients.

**Methods:**

The study included 40 post HCV-Hepatocellular carcinoma patients, 40 chronic HCV patients divided into 2 subgroups, 20 cirrhotic patients and 20 non-cirrhotic patients, and 40 apparently healthy subjects as a control group. Gene expression analysis for studied miRNAs was done using quantitative SYBR Green reverse-transcription Real-Time polymerase chain reaction (PCR).

**Results:**

We found that *Let-7a-1* gene expression was significantly downregulated in the serum of HCV-HCC patients than in HCV non HCC cirrhotic group and was significantly upregulated in the serum of liver cirrhosis patients than HCV non-cirrhotic group. *miR-143* and *miR-145* expressions were significantly downregulated in the serum of HCC patients than in control group and miR-143 was significantly downregulated in the serum of non-cirrhotic HCV patients than in control group.

**Conclusion:**

The downregulation of serum *let-7-a1*, *miR-143*, and *miR-145* gene expression may exhibit significant influence on the development of HCC in chronic HCV Egyptian patients and can be used as biomarkers for HCC diagnosis.

## Introduction

Egypt has the highest prevalence of HCV worldwide, which predisposes to high incidence of HCC in Egypt (Ziada et al., 2016). The need for new biomarkers with higher diagnostic accuracy is mandatory for early HCC diagnosis (Mancebo et al., 2018). The dysregulation of miRNAs expression is a key pathogenic mechanism in HCC development (Irshad et al., 2017). Among several miRNAs implicated in HCC, miR-143/145 cluster and let-7 family, were found to be downregulated in HCC compared to normal tissues (Gramantieri et al., 2007). Let-7 acts as tumor suppressor by suppressing growth signaling proteins (Chang et al., 2013). MiR 143/145 cluster contains two miRNAs, miR-143 and miR-145, they act as tumor suppressors by promoting cell cycle arrest (Iio et al., 2010). Both miRNAs were down-regulated in HCC (Tvingsholm et al., 2018). The dysregulation of miR-143/145 in tissues is one of the early events in cancer development (Wang et al., 2014). 

Accordingly, the present work aimed to identify the role of *miRNA let-7 a-1*, *d-1*, *f -1* and *miR143/ 145* cluster’s gene expressions in sera of chronic HCV, liver cirrhosis and HCC Egyptian patients.

## Materials and Methods

The study was approved by the Institutional Review Board of Theodor Bilharz Research Institute (FWA 00010609), and informed consent was obtained from each participant, in accordance with the ethical standards of the ethics committee of our hospital and with the 1975 Helsinki declaration and its later amendments.

*Patients*

This case-control study was carried out in the period from March 2017 to June 2017. One hundred and twenty subjects participated in our study. All HCC and chronic HCV patients were recruited from the tropical medicine department, Kasr Al-Ainy Hospital, Cairo University, and tropical medicine department, Theodor Bilharz Research Institute. All patients were subjected to full history taking, full clinical examination. The study included three groups; HCC, chronic HCV and control group. HCC Group included 40 newly diagnosed patients with HCC on top of liver cirrhosis due to HCV infection (28 males and 12 females with a mean age of 59.3 ±5.2). The diagnosis was established depending on alpha-fetoprotein (AFP) and specific imaging pattern by triphasic abdominal CT scan according to European Association for the Study of the Liver guidelines. Chronic HCV group included 40 patients with chronic HCV infection divided into 2 subgroups 20 cirrhotic patients and 20 non-cirrhotic patients (28 males and 12 females with a mean age of 60±4.9). HCV was diagnosed by positive HCV antibody and liver cirrhosis was diagnosed by abdominal ultrasound showing features of liver cirrhosis. The Control group: included 40 apparently healthy subjects with no history of liver disease, alcohol abuse or diabetes. Control group showed normal liver function tests and were negative for both HBsAg, HCV Ab tests (28 males and 12 females; with a mean age of 59.3± 5). Patients with the following criteria were excluded: chronic HBV infection, patients who received antiviral therapy for HCV infection or any loco-regional therapy for HCC. 

*Laboratory Investigations*

10 milliliters venous blood were withdrawn from all subjects and divided as follows: A) 3 milliliters were collected on a serum vaccutainer tube; the separated serum was used for determination of routine laboratory investigations, alpha fetoprotein and hepatitis markers. B) Three milliliters were collected on a sodium citrate vaccutainer tube; the separated plasma was used for determination of Prothrombin concentration (PC) and International Normalized Ratio (INR). C) Two milliliters were collected on an ethylene diamine tetraacetate “EDTA” vaccutainer tube, for complete blood count (CBC). 

D) Two milliliters were collected on a serum vaccutainer tube for RNA extraction. The routine laboratory investigations were assessed in the form of: serum total bilirubin, direct bilirubin, alanine aminotranseferase (ALT), aspartate aminotransaminase (AST), total protein, serum albumin, alkaline phosphatase (ALP), serum urea and creatinine, all assayed by the automated autoanalyzer Beckman Coulter AU 480. Serum AFP level was assayed using chemiluminescence by Advia Centaur CP instrument using kit purchased from Siemens Healthcare Diagnostics. Serum HBsAg and HCV Ab were assayed on Axsym auto analyzer using kits supplied by ABBOT. CBC, was done on CELL-Dyn 3700 using kits supplied by Spectra Group. PC and INR, were assayed on STAGO-STA Compact-ct using kits supplied by Diagnostica Stago. 

*Gene expression Analysis*

Of studied miRNAs in serum was done using quantitative SYBR Green reverse-transcription real-time PCR. Ce_miR-39_1 was used as an internal control and SNORD 68 was used as a housekeeping gene.

*Preparation of serum and miRNA extraction*

Two milliliters of blood were collected on a serum vacutainer tube and centrifuged 3,000 rpm for 5 min at room temperature. The supernatant was transferred to Eppendorf tubes which were stored at -80°C until RNA extraction. These samples were used for the determination of expression level of studied miRNAs, Ce_miR-39_1 and SNORD 68. RNA was extracted using the miRNeasy Kit (*Qiagen, Hilden Germany), using QIAcube extraction protocol according to the manufacturer’s procedure on QIAcube instrument (Qiagen, Hilden, Germany). RNA was quantified using the quawell q5000 UV-vis spectrophotometer (†Quawell Technology, USA).

*Quantitative SYBR Green reverse-transcription Real-Time polymerase chain reaction*

A total of 60 ng RNA per reaction was reverse transcribed using the mi-Script RT-II Kit (Qiagen) in a total reaction volume of 20 μL using thermal cycler Biometra Shared (†Biometra GmbH, Germany) according to the following conditions: 60 min at 37ºC., 5 min at 95ºC then 4°C on hold. Serum levels of studied miRNAs were assessed using the miScript SYBR Green PCR Kit (Qiagen), according to the manufacturer’s procedure. All the miRNA primers, were purchased from Qiagen. Sequences of all miRNAs were known using the miRbase (http://www.mirbase.org). Catalogue numbers of primers used are described in Table 1.

QuantiTect SYBR PCR reagent with miScript universal primer was used according to the manufacturer’s protocol (Qiagen). Quantitative PCR amplification and analysis were done on StepOneTM Real-Time PCR systems (Applied Biosystems, USA). 

The PCR reaction mixture included 10µL 2x QuantiTect SYBR Green PCR Master Mix (Qiagen), 2µL of 10x miScript Universal Primer, 2µL of 10x miScript target primer assay, 3μL RNase-free water, and then 3µL template complementary DNA in a total PCR reaction volume of 20 µL. Using the following thermal cycling program: incubation at 95^O^C for 15 min followed by 40 cycles of denaturation at 94^O^C for 15 sec, annealing at 55^O^C for 30 sec and extension at 70^O^C for 30 sec. After amplification, melting curve analysis was made and automatically the StepOne Real time-PCR systems calculated the negative derivative of the change in fluorescence and made a melting curve for each sample. The relative expression (fold change) in the miRNA expression level was calculated using the equation 2^-ΔΔCT^, using Qiagen Gene Globe 

Data analysis centre (Qiagen) after calibration with spike-in control and using SNORD68 as a normalization control.

*Statistical Methods*

 Quantitative data were summarized as mean ± SD when normally distributed and as median and (25^th^-75^th ^percentiles) if they were non-parametric. Differences between groups were detected using ANOVA with post hoc test, Kruskal Wallis and Mann Whitney tests as appropriate. Qualitative data were summarized as number and percentages and compared by Chi-square (*X*^2^) test. Receiver operating characteristic (ROC) curves were constructed for different miRNAs for discrimination of HCC and liver cirrhosis, with monitoring of the area under the curve (AUC) and calculation of sensitivity, specificity, and accuracy at different cut-off levels. All tests were two-tailed and considered statistically significant at a P-value <0.05. Statistical analysis was run on SPSS for mac, release 24 (IBM Corporation, Armonk, NY, USA).

## Results

The clinical and laboratory data are summarized in Tables 2-5. Upon comparing age and sex between different groups no significant difference could be detected.

*The laboratory data*

The median level of serum AFP showed significantly higher values in HCC group than in liver cirrhosis and HCV groups, (p <0.001 and <0.001 respectively), and also showed significantly higher values in the liver cirrhosis group than in the HCV group (p <0.001).

*Expression of miRNA in the four studied groups*

**Figure 1:**
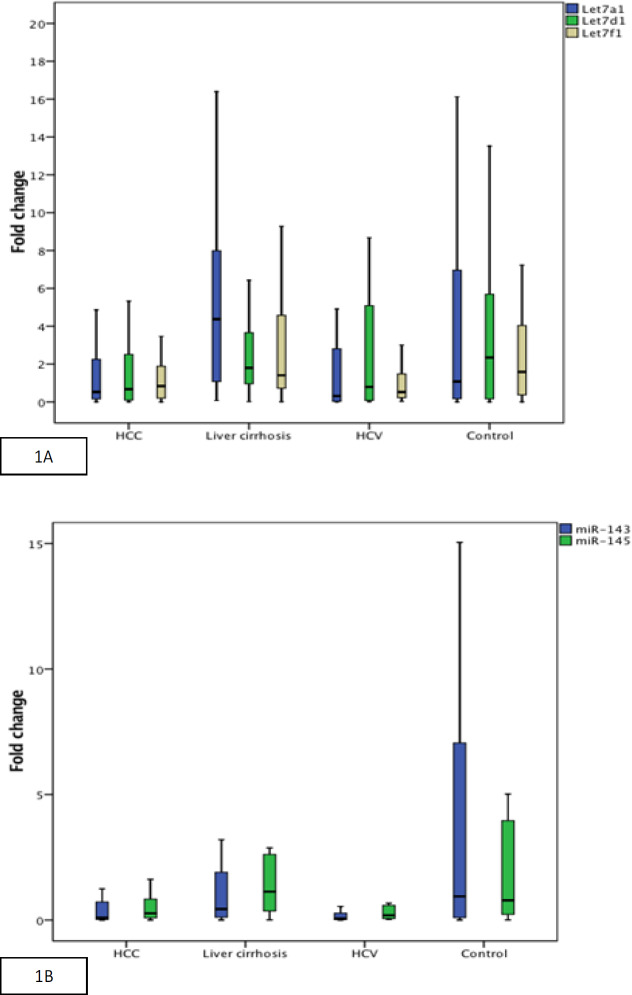
A, Box and whisker plot of Let7a1, Let7d1and Let 7f1 fold change among the four studied groups. B, Box and whisker plot of miR-143 and miR-145 fold change among the four studied group

Serum levels of studied miRNAs were investigated for their utility to detect HCV, liver cirrhosis and HCC. The levels of miRNAs were compared between patients and controls. Expression of *let-7a-1*, *miR-143*, and *miR-145* differed significantly between the 4 studied groups, (p=0.011,0.001 and 0.003 respectively). We found that the median values of fold change (FC) of Let-7a-1 were significantly lower in the serum of HCC patients than the liver cirrhosis group, (0.52 FC versus 4.37 FC) (p =0.003). The median values of FC of Let-7a-1 were significantly higher in the serum of liver cirrhosis patients than in HCV group (4.37 FC versus 0.31 FC) (p =0.003). Median values of FC of miR-143 and miR-145 were significantly lower in the serum of HCC patients than in the control group (p =0.002 and 0.006 respectively). Median values of FC of miR-143 were significantly lower in the serum of HCV patients than in control group (p = 0.002) (Table 6). The median values of the fold change of the studied miRNAs in the four studied groups are shown in Figure 1A, 1B. 

The median values of let-7-f-1 were significantly higher in HCC patients with BCLC stage 0 to A than stage B, (1.5 FC versus 0.1 FC) (p = 0.008). Studied levels of miRNAs gene expression didn’t differ significantly between groups with different degrees of liver fibrosis neither didn’t differ between different Child-Pugh classes.

It was found that median levels of miR-143 were significantly lower in smokers than non-smokers, (0.08 FC versus 0.28 FC) (p =0.01).

*Correlation between individual miRNAs and other parameters*

Let-7a1 showed significantly positive correlation with miR-let-7d1 (r= 0.455, p <0.001), let-7f1 (r =0.619, p <0.001) and miR-143 (r=0.643, p <0.001). Correlation between different markers and tumor characteristics yielded that AFP had significantly positive correlation with the largest diameter of the main tumor (r=0.583, p <0.001), and let-7d-1 showed a statistically significant negative correlation with the largest diameter of the main tumor (r = -0.371, p = 0.019). No correlation could be detected between different laboratory parameters and median values of* miRNA* expression. 

*Diagnostic Performance of miRNA*

**Figure 2:**
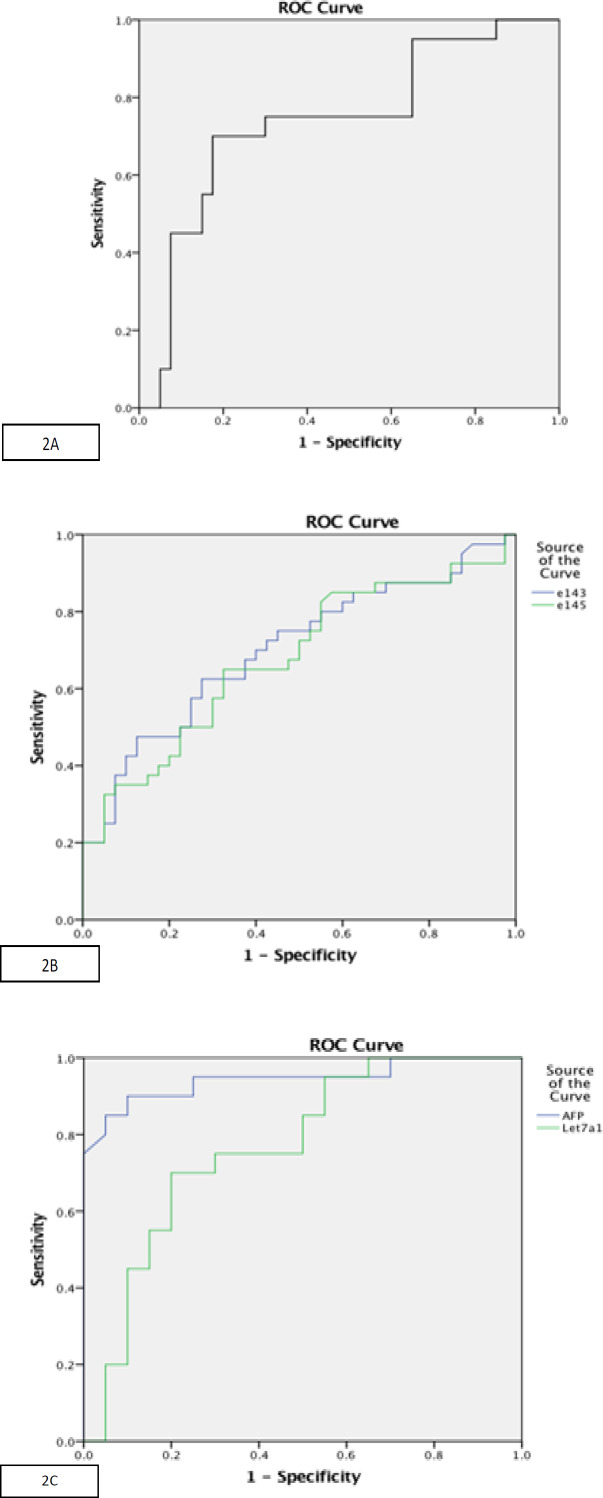
A, ROC curve for let-7a-1 to discriminate HCC from liver cirrhosis; B, ROC curve for miR-143&145 to discriminate HCC from controls; C, ROC curve analysis for AFP and let-7a-1 to discriminate liver cirrhosis from HCV

**Figure 3:**
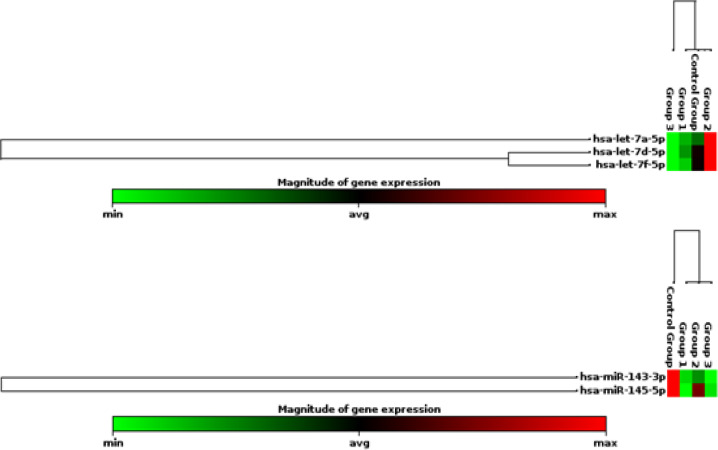
Heat Map of Studied miRNAs Expression; (Group1, HCC; Group 2, cirrhosis and Group 3, HCV).

ROC analysis was performed to evaluate the role of serum let-7a-1 as a potential diagnostic marker to discriminate between HCC and liver cirrhosis. At the cut-off value of ≤ 3.0, serum let-7a-1 showed sensitivity of 70% and specificity of 82.5% with AUC of 0.740 (p = 0.003, 95% CI 0.601- 0.879) (Figure 2A, Table 7). ROC analysis was performed to evaluate the usefulness of serum miR-143 as a potential diagnostic marker for HCC, at the cut-off value of ≤0.43, serum miR-143 showed sensitivity of 62.5% and specificity of 72.5% with AUC was 0.702 (p = 0.002, 95% CI 0.587- 0.817) (Figure 2B), (Table 8). ROC analysis was performed to evaluate the usefulness of serum miR-145 as a potential diagnostic marker for HCC (Figure 2B). At the cut-off value of ≤0.462, serum miR-145 showed sensitivity of 65% and specificity of 67.5% with AUC was 0.677 (p= 0.006, 95% CI 0.559- 0.795). ROC analysis was performed to evaluate the usefulness of serum let-7a-1 as a potential diagnostic marker for detection of liver cirrhosis development on top of HCV. At the cut-off value of ≥ 1.5, serum let-7a-1 showed AUC of 0.768 (p = 0.004) (Figure 2C). Figure 3 shows Heat map of studied miRNAs expression in the studied groups.

*Analysis of AFP as a marker and its combination with miRNAs*

ROC analysis for AFP was performed to differentiate HCC from liver cirrhosis, at the cut-off value of ≥ 132 ng/ml, AFP showed sensitivity of 82.5% and specificity of 100% with AUC of 0.971 (p < 0.001, 95% CI 0.937- 1.000). For combination of AFP and let-7a-1 a positive result of at least one marker (AFP ≥ 132 ng/ml and /or let7a-1 ≤3.0) was considered to indicate HCC and this combination improved diagnostic sensitivity to become 95% (Table 7). Regarding AFP ROC curve to differentiate cirrhosis from HCV, at the cut-off value of 10.0 ng/ml, serum AFP showed sensitivity of 70% and specificity of 100% with AUC of 0.944 (p < 0.001, 95% CI 0.868- 1.000). For analysis of combination of AFP and let-7a-1, a positive result of at least one marker (AFP and /or let-7a-1) was considered to indicate liver cirrhosis, which improved sensitivity to detect cirrhosis to become 95% (Table 9). 

**Table 1:** Target miRNA Sequences to be Studied and Amplified by qRT-PCR

Target MiRNA	Qiagen Catalog No.	Sanger Accession	Mature sequence
hsa-let-7a-5p	MS00031220	MIMAT0000062	UGAGGUAGUAGGUUGUAUAGUU
hsa-let-7d-5p	MS00003136	MIMAT0000065	AGAGGUAGUAGGUUGCAUAGUU
hsa-let-7f-5p	MS00006489	MIMAT0000067	UGAGGUAGUAGAUUGUAUAGUU
hsa-miR-143-3p	MS00003514	MIMAT0000435	UGAGAUGAAGCACUGUAGCUC
hsa-miR-145-5p	MS00003528	MIMAT0000437	GUCCAGUUUUCCCAGGAAUCCCU

**Table 2:** Clinical Data of the HCC, Liver Cirrhosis and HCV Groups

Item	HCC (n=40)	Cirrhosis (n=20)	HCV (n=20)	p
Smoking	23 (57.5%)	6 (30%)	4 (20%)	<0.001
Diabetes	14 (35%)	10 (50%)	4 (20%)	<0.001
Bilharziasis	19 (47.5%)	6 (30%)	4 (20%)	0.090
Family history of cancer	24 (60%)	3(15%)	5 (25%)	0.001

**Table 3:** Clinical Data of the HCC and Liver Cirrhosis Groups

Item	HCC	Liver cirrhosis	p
Ascites	23 (57.5%)	16 (80%)	< 0.001
Hepatic encephalopathy	18 (45%)	10 (50%)	0.001
Child Pugh Class	HCC	Liver cirrhosis	p
A	8 (20%)	3 (15%)	0.422
B	19 (47.5%)	7 (35%)	
C	13 (32.5%)	10 (50%)	

Data are presented as number (percentage)

**Table 4:** Laboratory Data of the Four Studied Groups

Parameter	HCC (n=40)	Cirrhosis (n=20)	HCV (n=20)	Control (n=40)	p
PC (%)	71.75 (59-80)	53* (37-72)	99.5*† (96-100)	97.5*† (93.25-100)	<0.001
INR	1.31 (1.2-1.5)	1.5 (1.3-1.97)	1.005*† (1.0-1.09)	1.03*† (1-1.09)	<0.001
ALT (IU/l)	55 (46-79.5)	46 (18.5-95)	28.5*† (15-45.5)	10*†‡ (10-16.75)	<0.001
AST (IU/l)	64.5 (46.5-93)	59.5 (33-116)	27.5*† (20.25-38.75)	16.5*†‡ (12.25-26)	<0.001
Albumin (g/dl)	2.82 (2.52-3.0)	2.75 (2.42-3.0)	4.2*† (3.9-4.5)	4.1*† (3.9-4.3)	<0.001
TBIL (mg/dl)	2.0 (1.0-3.9)	2.1 (1.05-6.02)	0.5*† (0.37-0.6)	0.75*† (0.5-0.9)	<0.001
DBIL (mg/dl)	0.61 (0.32-2.27)	0.7 (0.27-3.57)	0.115*† (0.1-0.2)	0.1*†‡ (0-0.1)	<0.001
AFP(ng/ml)	271 (141.75-506.25)	15.5 (9.1-63.25) *	4 (3 - 5.6) *†	----	<0.001

Data are presented median (25^th^ -75^th^ percentiles); *significant difference from the HCC group; † significant difference from the liver cirrhosis group, ‡ significant difference from HCV group

**Table 5:** Clinicopathological and Radiological Data of HCC Group

Parameter	Value	
Largest diameter of main tumor (cm)	< 3cm	16 (40%)
	3-5cm	14 (35%)
	>5cm	10 (25%)
BCLC staging	Stage Zero to A	11 (27.5%)
	Stage B	14 (35%)
	Stage C to D	15 (37.5%)

**Table 6:** The Median Values of Fold Changes of miRNAs among the Studied Groups

	Median values of fold changes				
	HCC (n=40)	Cirrhosis (n=20)	HCV (n=20)	Control (n=40)	p
Let-7a-1	0.526	4.37 *	0.31†	1.07	0.011
	(0.145-2.26)	(0.7- 8.27)	(0.034-2.88)	(0.16-7.03)	
Let-7d-1	0.673	1.79	0.79	2.34	0.28
	(0.0915-2.51)	(0.94-3.88)	(0.75-6.88)	(0.15-5.7)	
Let-7f-1	0.84	1.4	0.51	1.58	0.166
	(0.197-1.90)	(0.65-4.63)	(0.19-1.52)	(0.35-4.09)	
miR-143	0.089	0.44	0.06	0.94 *‡	0.001
	(0.0247-0.81)	(0.1-2.02)	(0.013-0.273)	(0.09-7.39)	
miR-145	0.265	1.13	0.19	0.80*	0.003
	(0.0832-0.85)	(0.27-2.74)	(0.06-0.6)	(0.21-4.2)	

All Data are presented median as (25th -75th percentiles); Results were expressed as fold change (FC); *significant difference from HCC group at P < 0.05; † significant difference from liver cirrhosis group at P < 0.05; ‡ significant difference from HCV group at P < 0.05

**Table 7:** Comparison of Receiver Operating Characteristic Curves for Serum AFP, let-7a-1, and Combination of both in HCC Versus Cirrhosis Group

Variable	Cut off	Sensitivity	Specificity	PVP	PVN	DA
AFP	132 ng/ml	82.5%	100%	100%	74.1%	88.3%
Let-7a-1	≤ 3.0	70%	82.5%	66.7%	84.6%	78.3%
AFP + Let-7a-1	One positive of two	95%	100%	100%	90.9%	96.6%

PVP, Predictive value of positive; PVN, Predictive value of negative; DA, Diagnostic accuracy.

**Table 8:** Comparison of Receiver Operating Characteristic Curves for Serum miR-143 and miR-145 in the HCC Group Versus the Control Group

Variable	Cut off	Sensitivity	Specificity	PVP	PVN	DA
miR-143	≤0.43	62.5%	72.5%	69.4%	65.9%	67.5%
miR-145	≤0.462	65%	67.5%	66.7%	65.9%	66.3%

PVP, Predictive value of positive; PVN, Predictive value of negative; DA, Diagnostic accuracy.

**Table 9:** Comparison of Receiver Operating Characteristic Curves for Serum AFP, let-7a-1, and Combination of both in Cirrhosis Versus HCV Group

Variable	Cut off	Sensitivity	Specificity	PVP	PVN	DA
AFP	10 ng/ml	70%	100%	100%	76.9%	85%
Let-7a-1	≥1.5	75%	70%	71.4%	73.7%	72.5%
AFP + Let-7a-1	One positive of two	95%	100%	95%	95.2%	95%

PVP, Predictive value of positive; PVN, Predictive value of negative; DA, Diagnostic accuracy.

## Discussion

Aberrant *miRNA* expression profiles have been associated with the development of many cancers (Wang et al., 2013). The present study showed that *let-7a-1* expression was significantly downregulated in the serum of HCC patients than liver cirrhosis group, suggesting that let-7a-1 downregulation is associated with HCC development on top of HCV-cirrhotic liver (p=0.003). The level of expression of let-7a-1 was significantly upregulated in the serum of liver cirrhosis patients than the HCV group (p=0.003). Let-7 miRNAs act as a tumor suppressor by their ability to stop cell proliferation by negatively regulating RAS levels through binding to the 3′-UTRs of the RAS mRNAs (Sun et al., 2013). Therefore, increased expression of *let-7* in human cancer cells decreases RAS protein levels and thus suppress cancer and HCC development.

Our results come in accordance with Gramantieri et al., (2007), they found let-7a1, d1, f1 and, miR-143 AND miR-145 were down-regulated in HCC tissues compared with non-tumorous tissue, HCC was arisen on top of HCV and HBV. Similarly, Shi et al., (2017) in their study which was done on HCC tissues, found that the expression levels of *let-7a-1* were lower in HCC tissues when compared with non-tumorous tissue (p<0.05) and the expression level of let-7a-1 was higher in liver cirrhosis tissues than HCC tissues (p < 0.05).

We found that the expression level of *miR-143* and *miR-145 *were significantly downregulated in the serum of HCC patients than in control group, (p=0.002 and 0.006 respectively). Our results came in accordance with Mourad et al., (2018) who found that serum levels of miR-145 were significantly lower in the post-HCV-HCC group than in both chronic HCV and liver cirrhosis groups (p < 0.01). Similarly, Elemeery et al., (2017) found that miR-145 was significantly downregulated in serum of post-HCV HCC patients, in comparison to healthy controls (p<0.0001). Also, Zhang et al., (2017) found that serum miR-143 significantly decreased in the HCC patients compared to the control group (p < 0.05). Zhao et al., (2018) found in their study on 85 post-HBV HCC patients and a control group that serum *miR143* and *miR145* expression were significantly downregulated in HBV-induced HCC compared with normal control (p<0.01), which comes in accordance with our study results. We found a reduction in the expression level of *miR143/145 miRNA* cluster as the disease progresses from liver cirrhosis to HCC. which means that the down-regulation of the miR-143/145 cluster is greatly linked to the progression of the hepatocarcinogenesis. miR-145 performs its tumor-suppressive role by suppression of cell proliferation through targeting epidermal growth factor receptor (Mataki et al., 2016). It was reported that RAS signaling caused repression of the miR-143/145 by activating Ras-responsive element-binding protein 1 (RREB1) in many cancers. In turn, miR-143/145 targeted RAS and RREB1, creating a feedback circuit of RAS signaling. The under-expression of miR-143 resulted in the loss of inhibition on *RAS* expression, leading to tumor cell proliferation (Kent et al., 2010). These findings together with the results of the previous studies suggest that circulating miR-143 and 145 could be used as biomarkers for the detection of post-HCV HCC. 

We found that miR-143, miR-145, and let-7a-1 were downregulated in the serum of HCV patients as compared to controls. Levels of miR-143 were significantly lower in the serum of HCV patients than the control group (p = 0.002). This suggests that HCV infection leads to the downregulation of these tumor-suppressive miRNAs, which control cell proliferation leading to the development of cirrhosis then further downregulation of these miRNAs in the cirrhotic liver contributes to carcinogenesis. 

Similarly, El-Guendy et al., (2016) found that serum let-7a-1 was significantly down-regulated in HCV samples (p <0.05) in their study on 50 HCV patients. On the contrary, the study done by Mourad et al., (2018) showed a signiﬁcant increase in miR-145 level in chronic HCV patients compared to the control group (p <0.01). Law et al., (2012) found a progressive reduction of miR-145 from cirrhosis to dysplastic nodules and ﬁnally HCC. The discrepancies between different reports may be attributed to the variation in the procedures used, starting from collection of samples, the differences in the detection techniques and methods of data analysis (Ura et al., 2009).

Furthermore, we investigated whether circulating levels of studied miRNAs could be used to discriminate HCC from cirrhotic and non-cirrhotic HCV patients as well as from the healthy controls. Our study showed that let-7a-1 may serve as potential negative predictor for discriminating HCC from liver cirrhosis and miR-143 and miR-145 may be used for discrimination of HCC from healthy controls. Thus circulating levels of let-7-a-1, miR-143 and 145 could be used as biomarkers for detection of HCV induced HCC with good diagnostic efficacy. Also, our findings revealed that let-7a-1 may serve as a potential marker for the detection of liver cirrhosis development on top of HCV. The combination of let-7-a1 and AFP improved the diagnostic accuracy of AFP to detect HCV induced HCC and HCV induced liver cirrhosis. 

Let-7a1 showed significantly positive correlation with let-7d1, let-7f1 and miR-143, which clarifies that let-7a-1, d-1, f-1 work with in the same network as tumor suppressor miRNAs with the same action of inhibiting RAS. Let-7d-1 showed a statistically significant negative correlation with the largest diameter of the main tumor. This suggests that let-7d-1 upregulation in HCC patients is associated with better prognosis than let7-d-1 downregulation, due to the role of let-7d-1 in tumor suppression. Also, let-7-f-1 was significantly higher in HCC patients with early BCLC stages than HCC patients with late BCLC stages which suggest the prognostic roles of these miRNAs in HCV-induced HCC. 

We can suggest that HCV infection leads to reduction in the level of expression of *let-7a-1* and *miR143/145* cluster which subsequently leads to the development of HCC.

In conclusion, the results of this study suggest that the downregulation of serum *let-7-a1*, *miR-143*, and *miR-145* expression may exhibit a significant influence on HCC development in chronic HCV Egyptian patients and can be used as biomarkers for HCC diagnosis to increase diagnostic accuracy when combined with AFP. Thus, it is mandatory to investigate the role of these miRNAs in different populations, with different HCV genotypes and other risk factors for HCC to assess the role of these miRNAs in HCC.
